# Analysis of Solar Irradiation Time Series Complexity and Predictability by Combining Kolmogorov Measures and Hamming Distance for La Reunion (France)

**DOI:** 10.3390/e20080570

**Published:** 2018-08-01

**Authors:** Dragutin T. Mihailović, Miloud Bessafi, Sara Marković, Ilija Arsenić, Slavica Malinović-Milićević, Patrick Jeanty, Mathieu Delsaut, Jean-Pierre Chabriat, Nusret Drešković, Anja Mihailović

**Affiliations:** 1 Faculty of Agriculture, University of Novi Sad, Dositej Obradovic Sq. 8, 21000 Novi Sad, Serbia; 2 Faculty of Sciences and Technology, University of La Réunion, Laboratoire d’Energétique, d’Electronique et Procédés, 15 Avenue René Cassin, Sainte-Clotilde, 97715 La Réunion, France; 3 Department of Civil Engineering and Architecture (DICA), University of Trieste, via Valerio 6/1, 34100 Trieste, Italy; 4 ACIMSI—Center for Meteorology and Environmental Modeling, University of Novi Sad, Dositej Obradovic Sq. 7, 21000 Novi Sad, Serbia; 5 Faculty of Sciences, Department of Geography, University of Sarajevo, Zmaj from Bosnia 33–35, 71000 Sarajevo, Bosnia and Herzegovina

**Keywords:** solar irradiation at La Reunion, Hamming distance, Kolmogorov measures, solar power, Kolmogorov time, solar irradiation predictability

## Abstract

Analysis of daily solar irradiation variability and predictability in space and time is important for energy resources planning, development, and management. The natural variability of solar irradiation is being complicated by atmospheric conditions (in particular cloudiness) and orography, which introduce additional complexity into the phenomenological records. To address this question for daily solar irradiation data recorded during the years 2013, 2014 and 2015 at 11 stations measuring solar irradiance on La Reunion French tropical Indian Ocean Island, we use a set of novel quantitative tools: Kolmogorov complexity (*KC*) with its derivative associated measures and Hamming distance (*HAM*) and their combination to assess complexity and corresponding predictability. We find that all half-day (from sunrise to sunset) solar irradiation series exhibit high complexity. However, all of them can be classified into three groups strongly influenced by trade winds that circulate in a “flow around” regime: the windward side (trade winds slow down), the leeward side (diurnal thermally-induced circulations dominate) and the coast parallel to trade winds (winds are accelerated due to Venturi effect). We introduce Kolmogorov time (*KT*) that quantifies the time span beyond which randomness significantly influences predictability.

## 1. Introduction

Solar irradiation varies in space and time, and investigation of its variability is essential for planning, operation, and management of solar power converting energy from sunlight into electricity. Solar irradiance that comes to the ground surface on a horizontal plane is altered spatially and temporally on different scales by the ground orography as well as atmospheric conditions. Among those atmospheric conditions, clouds are the primary source of uncertainties in measurements and predictability of solar irradiance causing more randomness in either measured or predicted solar irradiance time series. Another problem that occurs in the power plant building and management is related to practice, i.e., the spatial coherence between solar resources at different locations. Many studies devoted to these problems are broadly considered and cited in [1,2].

In the past two decades, several information measures and methods of nonlinear dynamics have been employed to explain the complex and chaotic character of complex systems in nature including solar irradiation [3]. One of the information measures for quantifying the randomness of solar irradiation is the Kolmogorov complexity (*KC*). This measure and its derivatives—Kolmogorov spectrum, and Kolmogorov complexity spectrum highest value (*KCM*) [4]—provide information complementary to that of entropy, offering additional insight into the behaviour of complex systems. However, *KC* has rarely been used to analyze solar irradiation time series. The *KC* and its derivatives are essential to use because randomness is a key characteristic that has significant implications for solar radiation modelling and prediction. The objective of this study therefore is to investigate the complexity and predictability of half-day (from sunrise to sunset) solar irradiation time series of 11 stations at La Reunion (France), using information measures *KC*, *KC* spectrum, *KCM*, sample entropy (*SE*) and Hamming distance (*HAM*) as one of information distances and a proposed measure that is a combination of *KC* complexity and *HAM* distance (*KCHA*). In addition, we introduced the Kolmogorov time (*KT*) indicating time window within a time series where complexity remains nearly unchanged.

The purpose of this study is to investigate the degree of randomness and predictability of the half-day solar irradiation time series for La Reunion using five information measures, while the existing atmospheric phenomena were employed to explain the sources of that randomness and constraints in the predictability of behaviour of those time series. We point out how randomness in the measured half-day solar irradiation time series can affect the quality and reliability of the forecast and to what extent it is possible. Henceforth, we shall denote this quantity Kolmogorov time (*KT*), as it quantifies the time span beyond which randomness significantly influences predictability.

The study is organized as follows. Part 2 describes the Kolmogorov complexity and information measures derived from it (Kolmogorov complexity spectrum and its highest value), sample entropy and Kolmogorov time. Part 3 provides information on half-day solar irradiation data of 11 stations at La Reunion (France) data for the years 2013, 2014 and 2015 as well as station locations. Finally, part 4 includes both presentation of the results obtained and discussion of the nonlinear feature of half-day solar irradiation time series using: (1) Kolmogorov complexity measures, (2) Kolmogorov complexity spectrum, (3) sample entropy, (4) proposed interpolation measure as a combination of *KC* complexity and *HAM* distance (*KCHA*), (5) Kolmogorov time, and the predictability of daily values. The concluding remarks are given in part 5.

## 2. Method

### 2.1. Kolmogorov Complexity and Its Derivatives

#### 2.1.1. Kolmogorov Complexity

The Kolmogorov complexity [5] stems from algorithmic information theory, which has been increasingly employed for characterization of complex phenomena in many sciences. Let X denote solar irradiation time series and x its specific value. Kolmogorov complexity K(x) of an object x is the length, in bits, of the smallest program that can be run on a universal Turing machine U and that prints object x. While the complexity K(x) is not directly computable for an arbitrary object x, in practice it is approximated by the size of the ultimate compressed version of x [6,7]. More precisely, a binary object x is compressed, and the size of the compressed object is identified with Kolmogorov complexity K(x). Lempel and Ziv [6] suggested an algorithm (LZA) for calculating *KC* of a time series. We briefly describe the calculation of the *KC* complexity of a time series $X\left(x_{1},x_{2},x_{3},\ldots ,x_{N}\right)$ by the LZA algorithm. It includes the following steps. (1) Encoding the time series by creating a sequence S of the characters 0 and 1 written as s(i),i=1,2,…, N, according to the rule s(i) = 0 if $x_{i}<x_{t}$ or 1 if $x_{i}>x_{t}$, where $x_{t}$ is a threshold. The threshold is commonly selected as the mean value of the time series, while other encoding schemes are also available [8]; (2) Calculating the complexity counter c(N). The c(N) is defined as the minimum number of distinct patterns contained in a given character sequence. The complexity counter c(N) is a function of the length of the sequence N. The value of c(N) is approaching an ultimate value c(N) as N is approaching infinity, i.e., c(N)=O(b(N)) and $b\left(N\right)=N/log_{2}N$; (3) Calculating the normalized information measure $C_{k}\left(N\right)$, which is defined as $C_{k}\left(N\right)=c\left(N\right)/b\left(N\right)=c\left(N\right)N/log_{2}N$. For a nonlinear time series, $C_{k}\left(N\right)$ varies between 0 and 1, although it can be larger than 1 [9]. The LZA algorithm is described in [6,10] but in a condensed form. However, here we will elucidate this algorithm through an example that is more illustrative than examples used in the above-mentioned papers. Note that the pattern is a sequence in the coded time series which is unique and non-repeatable. 

Recognition of the patterns with the LZA algorithm can be described in the following way:

1. The first digit, no matter if 0 or 1, is always the first pattern;
2. Define the sequence S, consisting of the digits contained in already recognized patterns. Sequence S grows until the whole time series is analyzed;
3. Define sequence Q, needed to examine the time series. It is formed by adding new digits until Q is recognized as the new pattern;
4. Define the sequence SQ, by adding sequence Q to sequence S;
5. Form the sequence SQπ by removing the last digit of sequence SQ;
6. Now examine if sequence SQπ contains sequence Q;
7. If sequence Q is contained in sequence SQπ then add another digit to sequence Q and repeat the process until the mentioned condition is satisfied; and
8. If sequence Q is not contained in sequence SQπ the, n Q is a new pattern. Now the new pattern is added to the list of known patterns called vocabulary R. Sequence SQ now becomes a new sequence S, while Q is emptied and ready for further testing.

Now, let us consider a short example with the binary sequence 1011010. The procedure obtained by the LZA logarithm can be simply described in this way:

- The first digit is always the first pattern, which implies! R = 1
- S = 1, Q = 0, SQ = 10, SQπ = 1, Q 2/v(SQπ) ! R = 1 0
- S = 10, Q = 1, SQ = 101, SQπ = 10, Q 2 v(SQπ) ! 1 0 1
- S = 10, Q = 11, SQ = 1011, SQπ = 101, Q 2/v(SQπ) ! R = 1 0 11
- S = 1011, Q = 0, SQ = 10110, SQπ = 1011, Q 2 v(SQπ) ! 1 0 11 0
- S = 1011, Q = 01, SQ = 101101, SQπ = 10110, Q 2 v(SQπ) ! 1 0 11 01
- S = 1011, Q = 010, SQ = 1011010, SQπ = 101101, Q 2/v(SQπ) ! R = 1 0 11 010.

**Figure 1:**
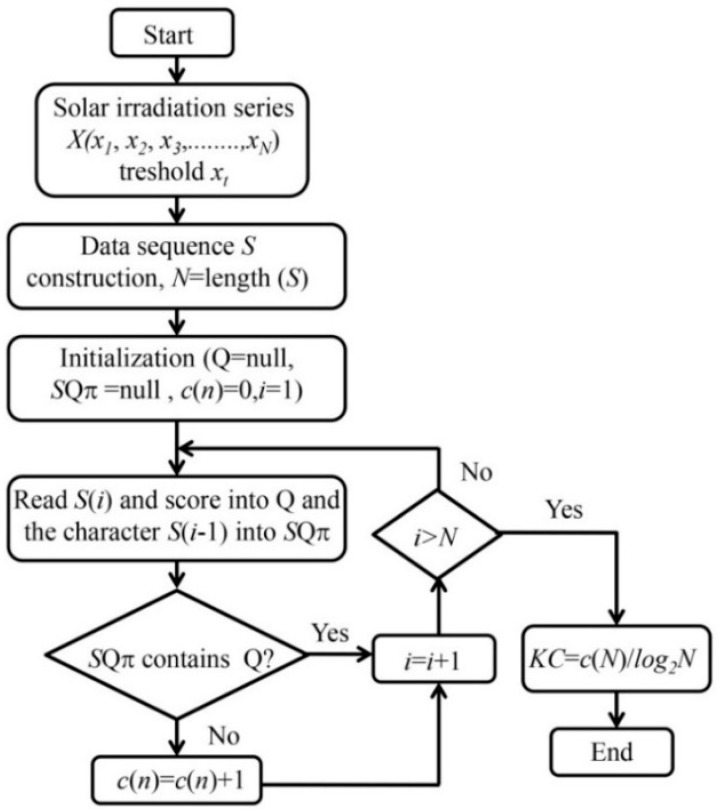
Flowchart for calculation of the Kolmogorov complexity (*KC*) using the Lempel-Zev algorithm (LZA).

Finally, vocabulary R consists of the patterns 1, 0, 11 and 010, which means that in this particular case the complexity counter is C(N) = 4. A flow chart for calculation of *KC* of a stream flow series $X\left(x_{1},x_{2},x_{3},\ldots ,x_{N}\right)$ using the LZA algorithm is shown in Figure 1. Despite the difficulties, which are often discussed, LZA algorithm is among the more accessible universal complexity estimators. However, complexity estimation using this algorithm usually amounts to performing the entire compression process and comparing inverse compression ratios as a measure of complexity. In fact, the simple Lempel Ziv partition contains enough data to estimate complexity without performing the entire compression encoding process.

#### 2.1.2. Kolmogorov Complexity Spectrum and Its Highest Value

**Figure 2:**
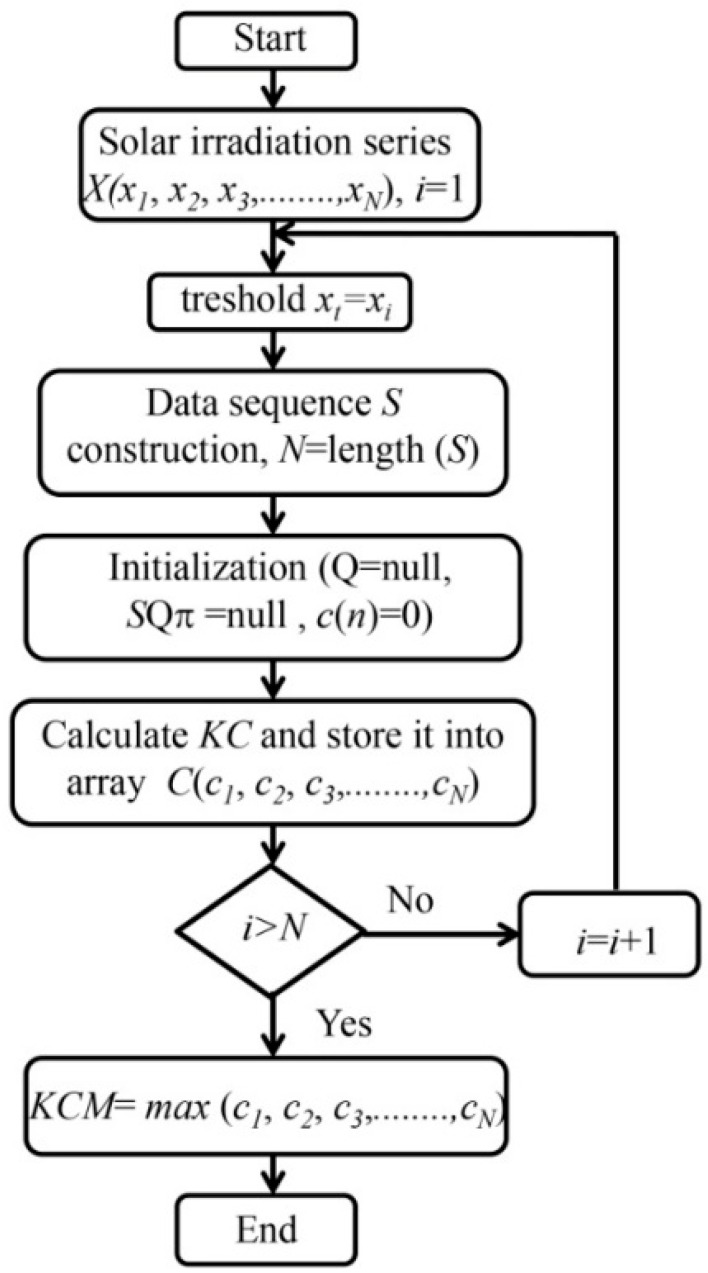
Flowchart for calculation of the Kolmogorov complexity spectrum and its highest value (*KCM*).

The Kolmogorov complexity of time series has some weaknesses. They are: (i) it cannot make a distinction between time series with different amplitude variations and that with similar random components; and (ii) in the procedure of conversion of time series into a binary string, its complexity is hidden in the rules of the applied procedure. Thus, in the definition of a threshold for a criterion for coding, some information about the structure of time series could be lost. In solar irradiation analysis, two measures were used: (i) Kolmogorov complexity spectrum (*KC* spectrum) and (ii) the highest value of *KC* spectrum, introduced by [4] who described the procedure for calculating the *KC* spectrum. Figure 2 schematically shows how to calculate the *KC* spectrum $C\left(c_{1},c_{2},c_{3},\ldots ,c_{N}\right)$ for solar irradiation time series $X\left(x_{1},x_{2},x_{3},\ldots ,x_{N}\right)$. This spectrum allows exploring the range of amplitudes in a time series that represents a complex system. The highest value $K_{m}^{C}$ as in this series, i.e., $K_{m}^{C}=max\left\{c_{i}\right\}$, is the highest value of Kolmogorov complexity spectrum (*KCM*), as seen in Figure 2. 

### 2.2. Hamming Distance Framework for Grouping the Stations Measuring Solar Irradiance

#### 2.2.1. Hamming Distance

The measure of the dissimilarity between two feature-vectors is an important topic from both a theoretical and practical standpoint. In that sense, an immense number of measures have been proposed during the last seven decades. Some of the most popular being: Hamming distance [11], Minkowski distance, Euclidean distance, Manhattan distance and Chebyshev distance [12]. Note, there exist many other measures created to be appropriate for a particular problem. However, all of them have a balance between advantages and disadvantages, which guarantees that any of them can be used as a general measure, either good or optimal, for all types of problems. In this study, we use the Hamming distance (*HAM*). Given x,y∈{0,1}, one way to measure how much they differ is the Hamming distance. If x,y∈{0,1} then HAM(x,y) is the number of bits on which *x* and *y* differ [13]. This measure is used in several disciplines including information theory, coding theory, and cryptography, telecommunication and many other scientific and practical fields. However, when we compare two sequences by computing *HAM* distance, by doing this comparison at the bit level for corresponding bits in each sequence, sometimes the distance may give a misleading answer [14]; this is shown in the next simple and illustrative example. Let us suppose we have two random sequences: SeqA = “0010110101000” and SeqB has the same length in which each corresponding bit is reversed (SeqB = “1101001010111”). Following the definition of *HAM* distance, the distance between these sequences would be maximal, i.e., they are unlike each other. However, in reality, we see that they are just a copy (“negative”) of each other. As pointed out by [15] if, in this case, bits represent an image, they are still the same, except that one is a “negative” copy of the other. Similar arguments are relevant for other distance measures. In this study, we will propose a simple information measure that combines the *HAM* distance and *KC* complexity.

#### 2.2.2. Measure Combining the Kolmogorov Complexity and Hamming Distance

**Figure 3:**
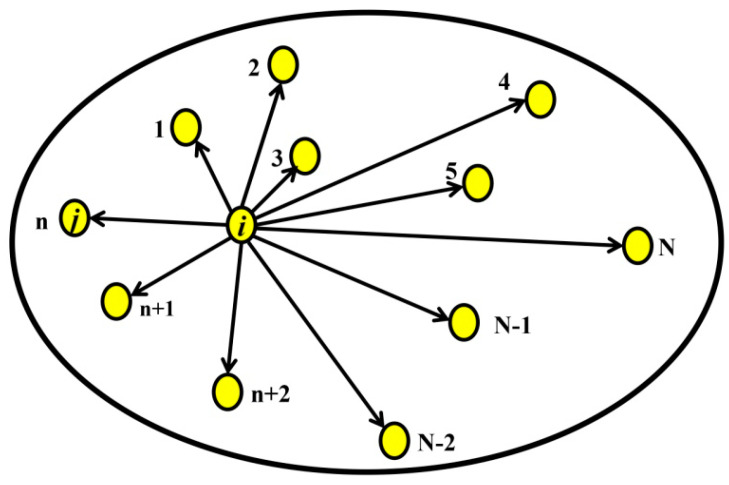
Towards the “spatial” Hamming distance. The yellow circles are objects, while the grey background bounded by the ellipse is a region containing the objects.

Let us have N objects in a region as it is shown in Figure 3. Then,

**Definition** **1.** *The “spatial” Hamming distance* $D_{reg,i}$ *is defined as* $D_{reg,i}=\sum_{i\neq j}HAM\left(x_{i},x_{j}\right),$ *where* i,j=1, 2, …,N*, while* $HAM\left(x_{i},x_{j}\right)$ *is the Hamming distance between objects* $x_{i}$ *and* $x_{j}$.

In this study, the objects represent stations measuring solar irradiance. Each of them is represented by a time series that carries information about this quantity for a certain time period. Therefore, in practice, we examine large pre-existing databases in order to generate new information. For that purpose, we use different information measures. One of them is the *HAM* distance that is described in the previous subsection. However, if we have more than two objects in some region (Figure 3) represented by the time series that generated the “spatial” Hamming distance $D_{reg,i}$ (*HAMR*), the natural question is why. We can think about this in the following way. Each object has its “own” time series, whose structure is the result of the local and regional influences coming from corresponding conditions. Thus, it seems that is reasonable to use an information measure that is a combination of the Kolmogorov complexity and Hamming distance representing local and regional influence, respectively. We will work with the normalized “spatial” Hamming distance (*HAMRn*), which is, for the *i*th object, defined as

(1) $$HAMRn_{i}=\frac{D_{reg,i}}{max\left\{D_{reg,i}\right\}}.,$$

**Figure 4:**
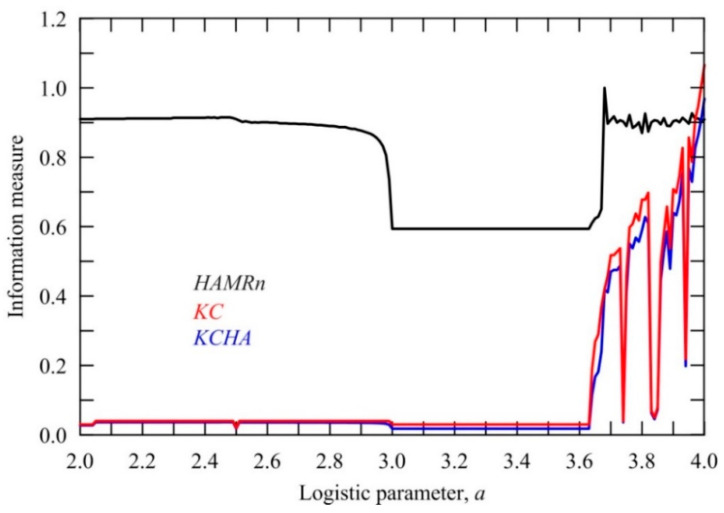
The “spatial” Hamming distance (*HAMRn*), Kolmogorov complexity (*KC*) and their product (*KCHA*) in dependence on the logistic parameter *a*.

For their combination, we use the product of those two quantities (*KCHA*) in the form.

(2) $$KCHA_{i}=\frac{1}{N}KC_{i}\cdot \sum_{j=1}^{N}HAMRn_{j},$$

where, j=1, 2, …,N, while KCHA∈[0,1]. The analysis using this information measure can be formally done for each set of objects using a time series that represents their evolution in time. Let us suppose that set is the set of logistic parameters a∈[0,4]. For all of them, we generate a time series from the logistic equation $x_{n+1}=ax_{n}\left(1-x_{n}\right)$, where n indicates the time. Results of calculations are summarized in Figure 4. From this figure, it is seen that objects with a>3.6, except in some intervals, have high values of *KCHA* because of the nature of the logistic equation. However, the measured time series situation is quite different since behind either lower or higher values of this information measure stands a background of physical, biological, etc. nature.

### 2.3. Calculation of Sample Entropy

The sample entropy (*SE*) quantifies regularity and complexity; it is an effective analyzing method of different settings that includes both deterministic chaotic and stochastic processes, predominantly operative in the analysis of physiological, hydrological, and radiative environmental interface signals involving a rather small amount of data [16]. In this measure, the filter r is an important parameter approaching zero with an infinite amount of data. For a finite number of data, its value usually varies between 10 and 20 percent of the time series standard deviation [17]. To calculate this measure for a time series $X=X\left(x_{1},x{,}_{2},\;\ldots ,x_{N}\right)$, it should follow these steps:

1. Create a set of vectors $X=X_{1}^{m},\;X_{2}^{m},\ldots ,\;X_{N-m+1}^{m}$ defined by $X_{i}^{m}=X\left(x_{i},x_{i+1},\;\ldots ,x_{i+m-1}\right)$, i=1,2,…,N−m+1;
2. The distance between $X_{i}^{m}$ and $X_{j}^{m}$, $d\left[X_{i}^{m},X_{j}^{m}\right]$ is the maximum of the absolute difference between their respective scalar components: $d\left[X_{i}^{m},X_{j}^{m}\right]=max_{k\in \left[0.m-1\right]}\left|x_{i+k}-x_{j+k}\right|$;
3. For a given $X_{i}^{m}$, the count number of j (1≤j≤N−m, j≠i) denoted as $B_{i}$, such that $d\left[X_{i}^{m},X_{j}^{m}\right]\leq r$. Then, for $1\leq i\leq N-m,\;B_{i}^{m}\left(r\right)=B_{i}/\left(N-m-1\right)$;
4. Define $B^{m}\left(r\right)$ as: $B^{m}\left(r\right)=\left\{\sum_{i=1}^{N-m}B_{i}^{m}\left(r\right)\right\}/\left(N-m\right)$;
5. Similarly, calculate $A_{i}^{m}\left(r\right)$ as 1/(N−m−1) times the number of j (1≤j≤N−m, j≠i); such that the distance between $X_{j}^{m+1}$ and $X_{i}^{m+1}$ is less than or equal to r. Set $A^{m}\left(r\right)$ as $\left\{\sum_{i=1}^{N-m}A_{i}^{m}\left(r\right)\right\}/\left(N-m\right)$. Thus, $B^{m}\left(r\right)$ is the probability that two sequences will match for m points, whereas $A^{m}\left(r\right)$ is the probability that two sequences will match m+1 points;
6. Finally, define: $SampEn\left(m,r\right)=lim_{N\to \infty }\left\{-ln\left[A^{m}\left(r\right)/B^{m}\left(r\right)\right]\right\}$ which is estimated by the statistics: $SampEn\left(m,r,N\right)=-ln\left[\frac{A^{m}\left(r\right)}{B^{m}\left(r\right)}A^{m}\left(r\right)\right]$.

### 2.4. Predictability and Kolmogorov Time

The *KC* complexity and its derivatives are essential to use because randomness is a crucial characteristic that has significant implications for solar irradiation modelling and prediction. Another complementary measure important for solar irradiation analysis is the Lyapunov time as the inverse of the largest Lyapunov exponent because it can detect the presence of deterministic chaos in complex systems (also in radiative processes) and quantify the predictability of potential future outcomes [18]. This time is used for predicting the time series. This is because the chaos theory generally deals with “irregular behaviour complex system that is generated by nonlinear deterministic interactions with only a few degrees of freedom, where noise or intrinsic randomness does not play an important role” [19]. Therefore, the Lyapunov time can overestimate the time for which the prediction of time series is reliable. Consequently, we introduce a correction of this time on randomness, which we call the Kolmogorov time (*KT*). It is estimated to be proportional to its randomness, i.e., KT=1/KC (in time units, second, hour or day, etc.). It could be stated that the *KT* designates the size of the time window within time series where complexity remains nearly unchanged.

**Figure 5:**
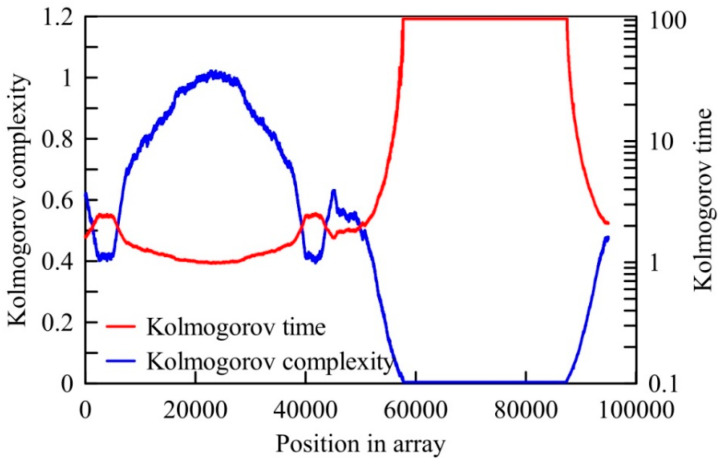
Towards an explanation of the Kolmogorov time (*KT*).

Let us visualize the meaning of the Kolmogorov time by the computational experiment using the logistic equation (see Section 2.2.2). Using this equation we created a time series of size N= 100,000 samples and logistic parameter a changing as

(3) $$a=3.75+0.25\sin{\left(2\pi \frac{i}{N}\right)}_{,}\;$$

where i is the sample position in the time. Using the window of 5000 samples long, we have calculated running average complexity, which is visualized in Figure 5. As shown in this figure, the *KC* is ranged in the interval (0, 1). Corresponding values of *KT* are also shown in the same figure. Simple inspection of this figure shows that *KT* is mostly located in the interval (1, 3) of time unit (TU). This means that the randomness is so high that we cannot reliably predict the behaviour of this time series. In contrast to that, for samples between 57,000 and 87,000, because of their low complexities, their *KT* is for an order of magnitude higher. The presence of the higher randomness in a time series may significantly reduce the predictability time of physical quantity we deal with. For example, values of *KC* for the solar irradiation time series is on average between 0.8 and 0.9. However, those values can be much lower because of lower randomness. Thus, if *KC* → 0 (low randomness) implies that the Kolmogorov time tends to ∞, then accurate long-term predictions are possible. Otherwise, if *KC* becomes considerably higher, accurate long-term predictions are not reasonable, but short-term ones can be made.

## 3. Data and Computations

### 3.1. Study Area

**Figure 6:**
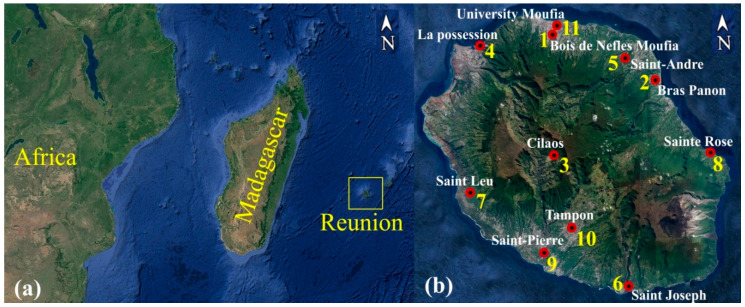
(**a**) La Reunion Island in the Indian Ocean and (**b**) the 11 stations used in the study.

The study area is Reunion Island (Figure 6a,b) which is a tropical oversea French department (2, 512 km²) located in the south-west Indian Ocean (21° S, 55° E) between Madagascar and Mauritius. The weather prevailing over the island is driven by the tropical synoptic wind (trade wind) and meteorological perturbations [ITCZ (The Intertropical Convergence Zone), convective clouds, tropical cyclone]. There are roughly two seasons: the dry period from May to October and the wet period from November to April. Moreover, La Reunion is a mountainous and volcanic island (highest altitude is 3070 m) with complex topography and cloud cover patterns. Location of stations measuring solar irradiance is shown in Figure 6, and their geographical coordinates are given in Table 1.

### 3.2. Solar Radiation Instrument

Solar radiation network (11 stations, see Table 1) which provide global horizontal irradiance (GHI) and the diffuse horizontal irradiance (DHI) at 1-min intervals are obtained using SPN1 sunshine pyranometer from Delta-T devices. Among the 11 stations, there are five stations from the National Electricity Company (EDF). This instrument is designed with an array of seven miniature thermopile sensors and a specific computer designed shading pattern which allows measuring the direct and diffuse components of incident solar radiation at the same time. This pyranometer has a spectral response set between 400–2700 nm with a solar radiation measurement range of 0 to >2000 W/m² and overall accuracy of ±10 W/m². There are analogy voltage outputs or digital RS232 signal, and the instrument is equipped with an internal heater. Its response time is 0.1 s and the zero off-set response is <3 W/m². The pyranometer’s resolution is 0.6 W/m² with non-stability <1%. The directional response of SPN1 is ±20 W/m², and the spectral sensitivity is ±10% (0.4–2.7 mm). Its temperature response is ±1%. The achievable uncertainty for SPN1 is 5% [20].

### 3.3. Short Description of Solar Irradiation Time Series

**Figure 7:**
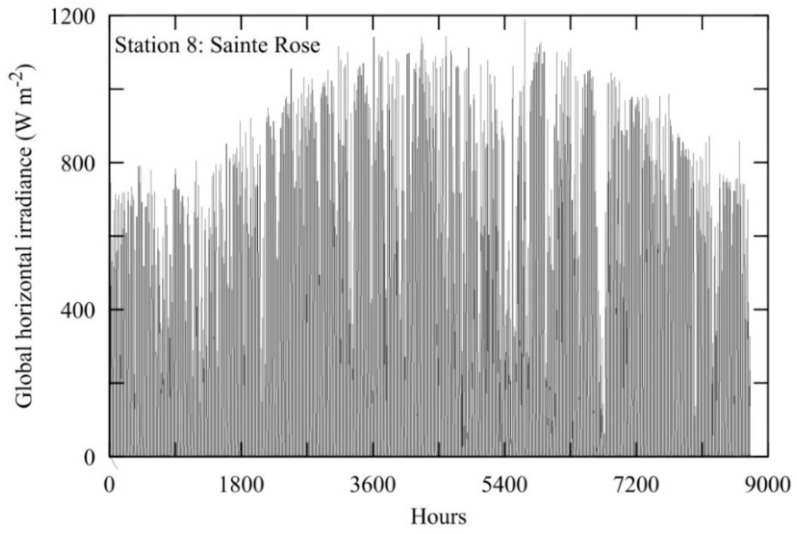
Time series of hourly global solar horizontal irradiance from sunrise to sunset for each day for Saint Rose for the period 1 June 2014–31 May 2015.

In this study, we analyzed the time series consisting of half-day solar irradiation which shows the highest *KC* complexity for all 11 stations (Figure 7 shows a typical graph of considered time series for station 8—Saint Rose). To extend the size of a time series, we were practically working with half-day sums of solar irradiation, although it shows some traces of daily periodicity. Half-day values consist of accumulated hourly global solar horizontal irradiance from sunrise to midday and from midday until sunset for each day. At the first part of the day they have a slightly higher value, but at the annual level, this feature is negligible. For stations 1, 2, 4, 5, 7, 9, 10 and 11 we used time series for the period 1 June 2013–31 May 2014, while for stations 3, 6 and 8 that period was 1 June 2014–31 May 2015. Because of the information measures we used and proposed in this study, we wanted to have as many stations as possible for analysis. We have made this choice because of the different time of installation of these stations and missing data. Since we dealt with half-day sums for global horizontal irradiation, there is a seasonality of the daily data from one year to year. On average the spatial intermittency pattern is nearly the same from year to year. Therefore, our results are consistent even with the different time period.

## 4. Results and Discussion 

**Figure 8:**
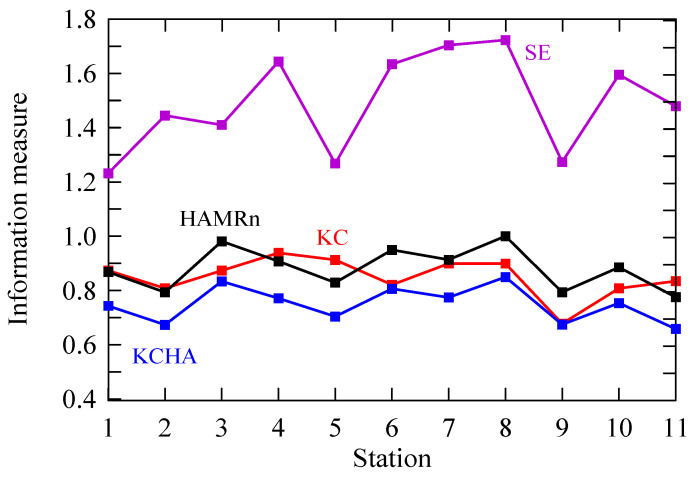
Values of information measures for half-day solar irradiation time series of 11 stations at La Reunion (France): Kolmogorov complexity (*KC*), sample entropy (*SE*), normalized “spatial” Hamming distance (*HAMRn*) and product of *HAMRn* and *KC* (*KCHA*).

**Figure 9:**
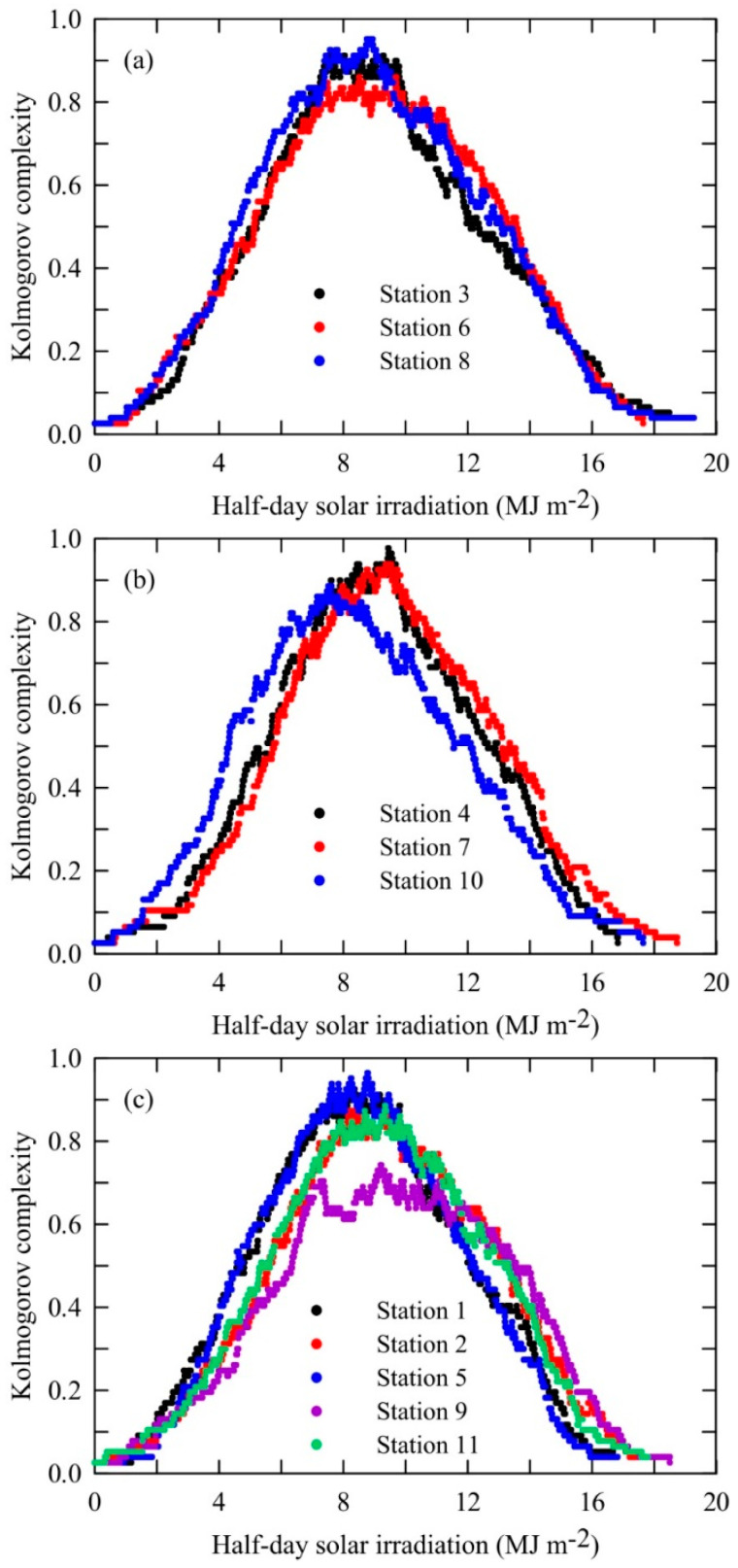
Kolmogorov complexity spectra of half-day solar irradiation time series of 11 stations at La Reunion (France). (**a**) stations 3, 6 and 8; (**b**) stations 4, 7 and 10; (**c**) stations 1, 2, 5, 9 and 11.

The Kolmogorov complexity measures applied in this study shed additional light on the complex behaviour of solar irradiation time series, complementing the usual entropy approaches commonly found in the literature. The *KC*, *KCM* and *SE* values of half-day solar irradiation data are given in Table 2 and Figure 8, while the Kolmogorov complexity spectra are depicted in Figure 9. Table 2 and Figure 8 shows that the *KC* values for all half-day solar irradiation time series are ranged in a wide interval (0.678; 0.938). This is expected for the island-like La Reunion because of prevailing weather that is mostly driven by features of mountains, the tropical synoptic wind and meteorological perturbations like, for example, convective clouds and a tropical cyclone. Orography and these phenomena introduce less or more randomness in half-day solar irradiation time series. A simple inspection of the KC complexity in Table 2 indicates the existence of two groups of values: (1) higher than 0.85 (stations 4, 5, 7, 8, 3 and 1—in the yellow boxes) and (2) less than 0.85 (11, 6, 10, 2 and 9—in the blue boxes), where stations are ordered in a decreasing sequence for both groups.

Looking at Figure 8 and Table 2, it is seen that the stations from the first group are located at lower altitudes (33; 230 m) with the exception of station 3, which is placed at 1213 m. Additionally, all stations are somewhat distant from the island coast (1895; 19,275 m), not including stations 4 (193 m) and 8 (384). The second group of the stations is also situated at the lower altitudes (38; 558 m) but relatively close to the ocean (573; 8664 m). Roughly, the first group exhibits higher mean slope (ratio of altitude to the distance from the sea) with 8% compared to the second group with 5%. The highest *KC* values are for stations 4 and 5, which reflect the local impact of half-day solar irradiation time series compared to the regional one, which could be concluded for all other stations. However, because of the entanglement of many factors, we cannot precisely say which dominant local factor is. What is more, by *KC* we are not able to determine, even approximately, how significant the local or regional impacts are on this information measure. There exist many either local or regional factors influencing variability of surface solar irradiances increasing/decreasing the *KC* of the half-day solar irradiation time series: the seasonal variations of large-scale circulation and cloud properties (occurrence, height and reflectance), occurrence of stronger winds than the large-scale trade-wind along the coast at daytime (Venturi effect), development of diurnal thermally induced circulations, combining downslope and land-breeze at night, and upslope and sea breeze at daytime, etc. [1,21,22,23]. Let us note that the purpose of this study is to investigate the degree of randomness and predictability of the half-day solar irradiation time series for La Reunion using five information measures, while the aforementioned phenomena were employed to explain the sources of that randomness and constraints in the predictability of behaviour of those time series.

Figure 9 shows the Kolmogorov complexity spectra of half-day solar irradiation data. Inspection of these figures reveals that the station with the highest *KCM* is station 4 (La Possession), while the lowest one is for station 9 (Saint-Pierre). From Table 2 it can be seen that the distribution of stations regarding the order of *KCM* is the same as those grouped pursuing the order of *KC* values, i.e., (1) higher than 0.90 (stations 4, 5, 7, 8, 1 and 3) and (2) less than 0.90 (2, 10, 11, 6 and 9), where, for both groups stations are ordered in a decreasing sequence. We can ask ourselves: What does this spectrum recommend? It gives information about the randomness of each amplitude in the solar irradiation time series using all information, i.e., all samples representing the time series.

Table 2 and Figure 8 depict values of the sample entropy (*SE*) for considered stations. This is an information measure presented in a time series, which is unbiased and less dependent on data. In the computing procedure we have used the following parameters: (ii) embedding dimension (*m* = 2), tolerance (*r* = 0.2) and time delay (*τ* = 1). From Figure 8 it can be seen that *SE* follows trends of *KC* for stations except for trends between stations 1–2, 2–3 and 10–11. Higher values of *SE* support the conclusion about less regular half-day solar irradiation time series in the considered periods.

The Hamming distance is an essential measure for detecting the errors in transmission of information suggested by [11] as was already mentioned. However, in addition to this application, its use is essential in the investigation of, e.g., ranked variables of entities coded by numerical symbols. It merely counts the number of differences between the two sequences. Therefore, the distances are unaffected by the distance of the object from the origin. The *HAM* is frequently used, for example, in evaluating the diversity of genomic [24,25,26] and other related data [27].

Figure 8 and Table 3 depict the normalized “spatial” Hamming distance (*HAMRn*), and product of *HAMRn* and *KC* values (*KCHA*) of half-day solar irradiation time series of 11 stations at La Reunion (France). If we anticipate, as we did in this study, then *HAMRn* indicates a measure of the regional impact on the randomness in considered time series contrary to the *KC* measure representing a local impact. Looking at Figure 8 it is possible to see sources of the randomness in time series: (1) prevailing conditions governed by the regional impact (*HAMRn* is greater than *KC* in stations 3, 6, 8, 9 and 10—in the yellow boxes), (2) prevailing conditions governed by of the local impact (*KC* is greater than *HAMRn* in stations 5 and 11—in the blue boxes) and (3) prevailing conditions when both local and regional impacts are nearly the same such as in stations 1, 2, 4 and 7—in the green boxes). We can notice some consistency with the regional and local average flow pattern [21,28]. Assigned stations in group 1 correspond to stations mainly located in the windward side of the island where the trade wind flow is splitting by the Fournaise Volcano. Along the Northeast coast (stations 5 and 11), the local streamline pattern is driven by the guided accelerating flow over regular slope at essentially the same altitude (~100 m). The remaining stations in group 3 are located where the flow is a combination of thermally induced flow (sea-breeze effect) and splitting flow downstream group 1 [21]. Let us note that trends of *SE* and *KCHA* in Figure 8 have a common feature. Namely, after station 4, for both information measures, they have the same tendency.

**Figure 10:**
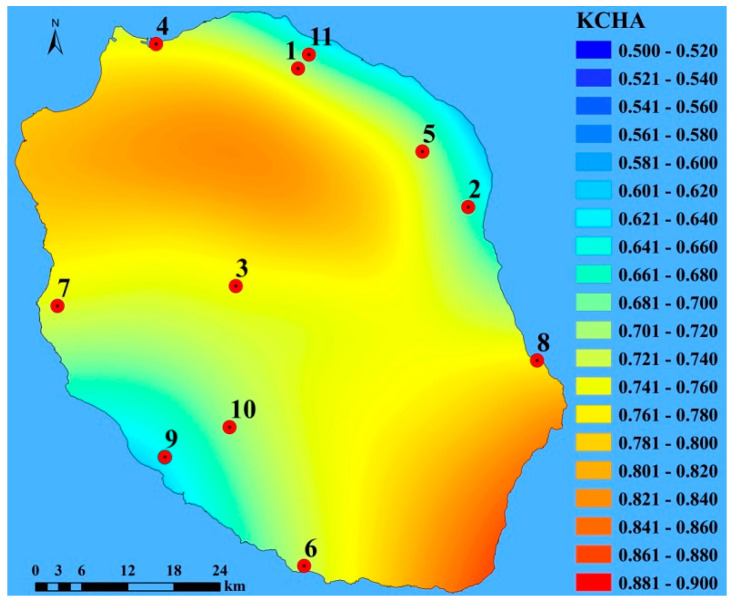
The spatial distribution of *KCHA* measure of half-day solar irradiation time series of 11 stations at La Reunion (France).

Using Equation (2), we calculated *KCHA* for all stations by merging both local and regional impact on time series. For each station, the order of this quantity was always 8, 3, 4, 7, 6, 1, 5, 10, 11, 2, 9. The spatial distribution of *KCHA* is visualized in Figure 10 where the zones with higher and lower randomness in time series are clearly separated. It seems that this combined measure can recognize both impacts on the randomness of half-day solar irradiation—cloudiness and spatial coherence—between solar stations at different locations. Figure 10 can be considered as an “average picture” of randomness of half-day solar irradiation time series over La Reunion. We notice three regions with different randomness: (1) station 9 with low values (South-West); (2) stations (1, 11, 5, and 2) located on North-East side of the island with higher values comparing to region 1 and (3) stations (4, 7, 6, 3, and 8) which are located in the direction extending from the South-East to the North-West side of the island, where the high randomness is dominant. Station 10 is just on the brink between regions 1 and 3.

There is no doubt that the spatial distribution of randomness half-day solar irradiation time series is caused by the distribution of cloudiness over La Reunion on different time scales. Here, we will not deal with the specific synoptic details of the occurrence of cloudiness. We instead give a simplified picture of the clouds formation and their distribution, which explains the distribution of randomness of the considered time series shown in Figure 10. This is lengthily described in a paper [22]. We just shortly summarize sources of cloudiness affecting the island: (i) the advection of trade cumuli and large-scale cloud systems; (ii) local formation by convection as a result of the interaction between synoptic wind, local thermal winds and the orography (Figure 6b) and (iii) acceleration of trade winds, along the island coasts parallel to the synoptic wind direction, due to the Venturi effect when clouds tend to be blown away. Following this classification of sources of cloudiness over the island, we can make the following commentaries for Figure 10. The region with high *KCHA* values is extended in the direction South-East to North-West, because of the higher cloudiness caused by conditions described in (i)—(stations 8, 3 and 6) and (ii)—(stations 7, 4 and nearly 10, whose time series is also influenced by some other factors). The stations 1, 5, 9, 2, 11 have the lower randomness of half-day solar irradiation time series because of lower cloudiness due to the Venturi effect (see condition (iii)). Lesouef [21] has shown a blocking of the trade wind by the island over the south-eastern side of the island. The low value observed at station 9 could be linked to this particular flow process.

In the above analysis, we used the Kolmogorov complexity measures and sample entropy to investigate the randomness of solar irradiance in a region with different geological elements. We have found the signature of these factors in the results obtained by the Kolmogorov-based information measures. However, it will be useful to discuss how distance from the sea can qualitatively affect the *KC.* The highest *KC* values are obtained for stations 1, 3, 4, 5, 7 and 8 (Table 2). The *KC* values for the stations closest to the sea coast (4 and 8, as seen in Table 1) are affected by the sea-breeze effect, trade wind effect and cloud cover too. Further inspection of the *KC* values in yellow fields in Table 2 indicates that there is a correlation of the *KC* not only with distance to the sea but also with the slope (ratio of station altitude to its distance from the sea coast). That slope has an average value of 7.5% for stations 1, 3, 5 and 7. The slope causes orography forcing, which can significantly contribute to cloud cover formation and thus contributes to the variability of the solar irradiance reaching the ground. We can get similar results for the *KCHA* (Table 3). From Table 2 we can see that solar irradiation time series measured at station 5 (Saint-Andre; 6028 m away from the coast) exhibits the highest *KC* but the lowest *KCHA* values. This evidence could be because at this station the local factor prevails over the regional one. This station is a representative of the windward coast with a lot of rain due to air uplift and accordingly a generator of clouds formation. Station 3 (Cilaos) is under both impacts—local and regional exhibiting high values for the *KC* and *KCHA.* Apparently, the behavior of the information measures cannot always be explained by the slope of the measuring stations. However, it seems that the slope of the site could have an impact on *KC* and accordingly on *KCHA*.

Forecasting of solar radiation is an issue with exceedingly practical consequences since it is expected that solar energy will be one of the major contributors in the future global energy supply. Because of its changeable nature, it is very difficult to give reliable forecast information at the various time and spatial scales in dependence on the application. In choosing the model for this kind of forecast, like other sciences, scientists often apply a heuristic technique that could be defined as an approach to problem-solving that makes use of a practical method not guaranteed to be optimal or perfect (this word is used metaphorically), but sufficient either for the immediate goals or until a better approach is found. Note, where discovery an optimal solution is not possible or impractical, heuristic methods can be used to speed up the process of finding a satisfactory solution. The current status of forecasting solar irradiance for energy generation purposes is comprehensively reviewed, in regard to short-term forecasting (up to a few hours) and forecasts for up to two days primarily for use in practical applications, by [29].

**Figure 11:**
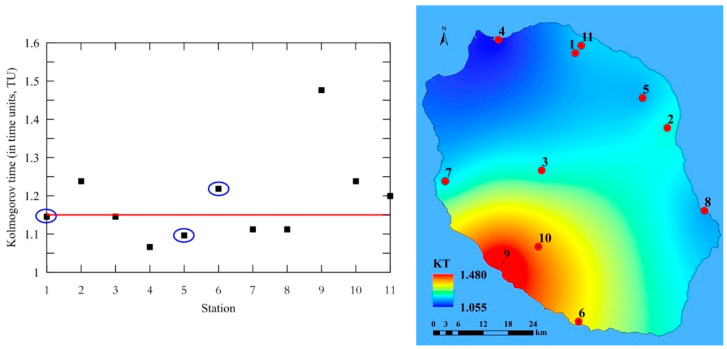
The Values of Kolmogorov time (*KT*) for half-day solar irradiation time series of 11 stations at La Reunion (France) (**left**) and its spatial distribution (**right**).

In this study, we do not intend to deal with the performance and quality of the solar radiation forecasting models. We rather point out how randomness in the measured half-day solar irradiation time series can affect the quality and reliability of the forecast and to what extent it is possible. Henceforth, we shall denote this quantity Kolmogorov time (*KT*), as it quantifies the time span beyond which randomness significantly influences predictability. The left panel of Figure 11 shows the predictability of half-day solar irradiation time series given by the *KT* in time units indicating the level of randomness in time series. The figure shows that the prediction effect for *KT* is extended between 1.1 and 1.5 TU. The stations having lower values of *KC* (and lower randomness) are placed above the red line (2, 6, 9, 10 and 11), while other stations (1, 3, 4, 5, 7 and 8) have *KT* values that are closer to one. This means that for these stations, solar radiation models cannot provide a reliable forecast. The right panel in Figure 11, showing the spatial distribution of KT, indicates its slower values, and thus less reliable solar radiation forecasts, going towards Northern, Eastern and South-Eastern regions of Le Reunion. Let us note that *KT* values in Figure 11 are calculated only on the basis of Kolmogorov complexity, which exposes the local influence (Table 2), and hence the stations rounded by a blue ellipse have a higher complexity and less *KT*. However, when regional influence is included, then the probability of better solar radiation forecasts for these stations increases. Let us note that the red line in Figure 11 is not a kind of absolute threshold for the predictability such that none of the models can guarantee any accuracy of forecasting. It simply indicates that for these stations the measured time series of the solar irradiance will have a domain of *KT* as shown in Figure 11. Depending on the performance of the models, they will be more or less successful in forecasting the solar irradiance.

Natural questions that arise are: (i) how can we incorporate the knowledge on the degree of randomness and predictability of the half-day solar irradiation time into the modelling tasks for solar irradiance? and (ii) can we use these results obtained in this study to determine the variables that will be included in the forecasting model, avoiding the irregular (chaotic) behaviour of the output time series? For clarity purposes in question (i), chaotic processes should not be confused with random processes because chaos does not imply randomness in any sense. Chaotic processes do not have any kind of distribution like random processes in nature. Furthermore, chaotic processes are perfectly deterministic while random processes are attached to some prior probabilities. Thus, the chaos is the paradigm in the field of nonlinear analysis which is used to describe the realms of nonrepeating and highly complex dynamic systems, which the solar irradiance is. If Lyapunov exponent (LE) is greater than 1, then the system under analysis is not a chaotic system but rather a stochastic one, and so we cannot make any predictions based on the Chaos Theory. If 0 < LE < 1, then it implies that there is chaos in the system. For practical purposes, the approximate period limit is usually computed, often called Lyapunov time, for accurate prediction since it is an inverse function of the maximal value of the LE. Thus, if LE → 0 implies that the Lyapunov time tends to ∞, then accurate long-term predictions are possible. Otherwise, if LE becomes considerably higher, accurate long-term predictions are not reasonable, but short-term ones can be made instead. However, the presence of the higher randomness (which can be detected from the analysis of measured solar irradiation time series) may significantly reduce the predictability time; (ii) Therefore, results obtained in this study can be used in the choice of equations and variables that could be included in a forecasting model. To conclude, let us note that the effect of randomness investigated by the proposed approaches could be, similarly, used for (i) establishing relationship between the effect of randomness and quality control processes used for the solar irradiance measurements and (ii) and evaluation of the quality of measurements after the application of range tests for data quality issues.

## 5. Conclusions

Half-day solar irradiation data from 11 gauging stations Le Reunion (France) are analyzed using the Kolmogorov complexity and related complexity measures (Kolmogorov complexity spectrum and its highest value), sample entropy, Hamming distance, a measure that combines Kolmogorov complexity with Hamming distance (*KCHA*) and Kolmogorov time. The following conclusions are drawn from this study:

1. Half-day solar irradiation time series exhibit a tendency in increasing the randomness in dependence on cloudiness, the position of the measuring station and the prevailing local or regional weather conditions. Such dependence seriously affects the short-term predictability of solar irradiation.
2. The values of *KC*, *KCM*, and *SE* of solar irradiation time series are ranged in a broader range that indicates pronounced local orographic and air flow impact on higher randomness.
3. Kolmogorov complexity spectra yield information about the randomness for each amplitude in the solar irradiation time series.
4. *KCHA* measure, as a combination of *KC* and *HAM* distance for each station by merging both local and regional impact on time solar irradiation series, can recognize both impacts on the randomness of solar irradiation time series—cloudiness and spatial coherence between solar stations at different locations.
5. The region with high *KCHA* values and corresponding randomness is extended in the direction South-East to North-West, because of higher cloudiness caused by (a) the advection of trade cumuli and large-scale cloud systems and (b) local formation by convection as a result of the interaction between synoptic wind, local thermal winds and the orography; the stations having lower randomness of solar irradiation time series because of lower cloudiness due to the Venturi effect.
6. Kolmogorov time (*KT*) quantifies the time span beyond which randomness significantly influences predictability. This means that for stations having the higher randomness of half-day solar irradiation time series, solar radiation models cannot provide a reliable forecast.
7. The relevance of the application of the suggested information measure can be synthesized as follows: (i) on the basis of the measured half-day solar irradiation time series it is possible to detect the level of its randomness and detect sources of that randomness, which is essential, for example, in building the solar powers and (ii) by Kolmogorov time, calculated from those time series, potential reliability of the solar radiation model forecast could be estimated.

## Figures and Tables

**Table 1:** Coordinates of stations measuring solar irradiance used in this study.

Number	Name of Station	Altitude	Longitude	Latitude	Distance from the Sea
		(m)	Degree East	Degree South	(m)
1	Boisde Nefles Moufia	336	55.476445	20.917310	3540
2	Bras Panon	32	55.682897	21.002649	1860
3	Cilaos	1213	55.47416	21.136158	19,725
4	La Possession	15	55.328967	20.930611	193
5	Saint-Andre	198	55.622433	20.962797	6028
6	Saint Joseph	38	55.619688	21.379077	573
7	Saint Leu	230	55.302332	21.200642	1895
8	Sainte Rose	33	55.793136	21.127324	384
9	Saint Pierre	85	55.451069	21.313922	1945
10	Tampon	558	55.507020	21.269277	8864
11	Saint Denis—University	85	55.483593	20.901460	1759

**Table 2:** Kolmogorov complexity (*KC*), sample entropy (*SE*), and the highest value in the Kolmogorov complexity spectrum (*KCM*) of half-day solar irradiation time series of 11 stations at La Reunion (France). The yellow boxes indicate the first group of stations (4, 5, 7, 8, 3 and 1), while the blue ones indicate the second group (11, 6, 10, 2 and 9).

Station	Information Measure		
Number	*KC*	*KCM*	*SE*
1	0.873	0.925	1.232
2	0.808	0.873	1.445
3	0.873	0.899	1.410
4	0.938	0.977	1.644
5	0.912	0.938	1.268
6	0.821	0.847	1.634
7	0.899	0.938	1.704
8	0.899	0.938	1.723
9	0.678	0.730	1.273
10	0.808	0.873	1.595
11	0.834	0.860	1.479

**Table 3:** The normalized “spatial” Hamming distance (*HAMRn*), and product of *HAMRn* and *KC* values (*KCHA*) of half-day solar irradiation time series of 11 stations at La Reunion (France). The colour of boxes: (1) *HAMRn* > *KC* (yellow), (2) *KC* > *HAMRn* (blue) and (3) *KC* ~ *HAMRn* (green).

Station	Information Measure	
Number	*HAMRn*	*KCHA*
1	0.868	0.758
2	0.792	0.640
3	0.980	0.856
4	0.906	0.851
5	0.829	0.779
6	0.949	0.756
7	0.912	0.820
8	1.000	0.899
9	0.793	0.538
10	0.885	0.715
11	0.775	0.646
